# A Model for the Stop Planning and Timetables of Customized Buses

**DOI:** 10.1371/journal.pone.0168762

**Published:** 2017-01-05

**Authors:** Jihui Ma, Yanqing Zhao, Yang Yang, Tao Liu, Wei Guan, Jiao Wang, Cuiying Song

**Affiliations:** 1 MOE Key Laboratory for Urban Transportation Complex Systems Theory and Technology, Beijing Jiaotong University, Beijing, China; 2 Department of Civil and Environmental Engineering, University of Auckland, Auckland, New Zealand; Beihang University, CHINA

## Abstract

Customized buses (CBs) are a new mode of public transportation and an important part of diversified public transportation, providing advanced, attractive and user-led service. The operational activity of a CB is planned by aggregating space–time demand and similar passenger travel demands. Based on an analysis of domestic and international research and the current development of CBs in China and considering passenger travel data, this paper studies the problems associated with the operation of CBs, such as stop selection, line planning and timetables, and establishes a model for the stop planning and timetables of CBs. The improved immune genetic algorithm (IIGA) is used to solve the model with regard to the following: 1) multiple population design and transport operator design, 2) memory library design, 3) mutation probability design and crossover probability design, and 4) the fitness calculation of the gene segment. Finally, a real-world example in Beijing is calculated, and the model and solution results are verified and analyzed. The results illustrate that the IIGA solves the model and is superior to the basic genetic algorithm in terms of the number of passengers, travel time, average passenger travel time, average passenger arrival time ahead of schedule and total line revenue. This study covers the key issues involving operational systems of CBs, combines theoretical research and empirical analysis, and provides a theoretical foundation for the planning and operation of CBs.

## 1 Introduction

The conflict between increasing traffic demand and a relatively lagged traffic supply is becoming increasingly prominent with the rapid development of economy and society and the accelerating process of urbanization. Traffic congestion, traffic environment pollution, traffic accidents, energy consumption and societal fairness problems are widespread in all large and medium-sized cities in our country. These difficult problems perplex the majority of urban travelers and government administration. To satisfy the increasing and changing traffic demand for citizens’ travel and minimize the network traffic load, Beijing proposed building a "public transport city" in 2009 and stated that managers should promote the construction of a green travel system that coordinates rail transit as the backbone, ground buses as the main body, walking and cycling. In recent years, CBs have come online in many large and medium-sized cities in China due to the rapid development of information and communication technology, particularly the widespread use of smartphone apps. The first CB was implemented in Beijing in October 2013; since December 2015, 287 CB lines have been implemented in Beijing. The average demand for a CB has increased from 200 in 2013 to more than 100,000 at present. With such a large-scale passenger demand for CBs, many large and medium-sized cities in China and other developing countries are now faced with determining how to scientifically plan, design, and operate CBs, improve the level of CB service, reduce the costs of operating CBs, facilitate passenger travel, and alleviate urban road congestion and smog.

The car-sharing problem, which is regarded as the earliest concept of CBs, was first conceived in Zurich in 1948 [1]. Kirby and Bhatt [2] analyzed ten cases of a subscription bus, which represents the implementation of the car-sharing idea in public transport. The authors provided guidelines on the planning, organization and operation of subscription bus services. Kirby and Bhatt [3] then noted seven main features of subscription bus services, such as a relatively large concentration of at least 50 fairly long trips with proximate origins or destinations, specialized organization operation management, constant adjustment of lines and schedules to meet demand, and guaranteed seats for personalized service. Based on the work of Kirby and Bhatt, a cost comparison between several types of subscription bus services was made by Bautz [4]. Bautz indicated that the least cost services were provided when the private carriers operated a subscription bus with the support of the government. McKnight and Paaswell [5] designed a subscription bus service in Chicago and indicated that it could help reduce the peak demand and deficit on certain commuter railroad lines. The authors discussed key elements of the subscription bus service that was successfully implemented in the public transport sector. Based on the travel demand of diversification and humanization, CBs came into being as a new travel mode. Shaheen et al. reviewed the development of CBs from the concept of "car sharing" and summarized the advantages of CBs [6], but CB networks and timetable modeling was not examined. Eiro et al. [7], Martinez et al. [8], and Lopes et al. [9] proposed methods based on clustering analysis and a multi-agent model to solve the network planning and timetable problems of a CB in Lisbon, Portugal. However, the dynamic and real-time of passenger demand were not considered in these articles. De Lorimier et al. [10] used the multi-hierarchical regression analysis method to determine the decisive effect of a CB system in Montreal on the effectiveness of vehicle use, providing a reference for the establishment or expansion of CB networks. Tao Liu [11] systematically reviewed the background and implementation of CBs, analyzed various stages of the CB operation planning process and summarized the shortcomings of CBs. The abovementioned studies regard CBs as a new mode of transportation, operation pattern and planning; comparative analyses between CBs and conventional bus are provided, but the stop planning and timetables of CBs are not involved. Based on passenger demand, operator characteristics, and social benefits, this article further examines the model and solution method for CB stop planning and timetables.

Bus stops design affects the alighting and boarding time of passengers and the dwell times of buses; thus, it is necessary to examine the stop design for CBs. In this section, we briefly review the relevant literature on bus stop design problems. Rodrigo Fernández [12] presented a microscopic model for the operation of public transport stops, mainly bus stops and light rail transit stations, from the perspective of traffic analysis. The authors showed that stop design should incorporate the possibility of allowing exact arrival and departure patterns for both vehicles and passengers. The proposed model provides more detailed information about stop operations. Gu W, Cassidy M J, and Li Y [13] considered curbside bus stops of the type that serve multiple bus routes and that are isolated from the effects of traffic signals. A Markov chain embedded in the bus queuing process was used to develop steady-state queuing models for two special cases of this stop type. The methods for the two special cases were used to derive a closed-form or parsimonious approximation model for general cases. To estimate the service time at a curbside bus stop, a compound Poisson service time estimation model (CPSTM) was proposed by Bian B, Zhu N, Ling S, et al. [14]. The model considered the interactions among arriving buses and the number of boarding and alighting passengers. Four different scenarios that occur in curbside bus stops were established, and the corresponding probability models were formulated based on the Poisson process. The abovementioned studies demonstrate that bus stop planning should consider the capacity, congestion, queuing, and serve time of bus stops. These modes and methods have provided great help in solving the stop planning and timetables of CBs.

Although there is little research on CB network design and timetables, it is worthwhile to provide a rather extensive review of conventional bus design models. Lampkin [15] and De Hsu [16] proposed methods for determining public transport networks and lines by classifying public transport lines and stops based on demand. Ceder, A. and N. H. M. Wilson [17] summarized research on public transport network design and proposed and solved a new model incorporating the benefits of passengers and operators. The proposed approach was easier to implement and required a smaller data set. Patanik [18] divided the route network design problem into two stages. First, a set of candidate routes competing for the optimal solution is generated. Second, the optimal set is selected using a genetic algorithm. Bielli et al. [19] described a method for transit line design using a genetic algorithm. In the proposed method, each iteration is based on the computational performance index of the distribution result, including the distribution demand in the existing network. To calculate the fitness function value, these indexes serve as input for each network in the multi-criteria analysis. The transit route network design problem was formulated as an optimization problem of minimizing the sum of the operating cost and the generalized travel cost by Agrawai [20]. In this study, two parallel genetic algorithm models are proposed. Through a case study, these models are tested with respect to computation time, speedup, and efficiency. Xiaolei M A, WU, YaoJan, et al. [21] proposed a data mining method capable of identifying travel patterns for individual transit riders using a large smart card dataset. The travel patterns could be beneficial to understanding the variability of urban travel behavior and facilitating network design. For more information regarding other models of network design and timetables, the reader is referred to Reference [22], Reference [23] and Reference [24].

The bus network problem always consists of a tremendous number of stops and stations, which makes the scale of the problem too large and intractable to obtain an optimal solution. GA is a flexible meta-heuristic algorithm that has shown outstanding performance in solving large-scale public transit problems. Mazloumi [25] solved the minimum cost solution using genetic algorithm and ant colony algorithm. The experiments indicated that optimal solution were obtained. A large city public transport network design problem consisting of a complex network topology, multiple modes and many-to-many travel demand was solved with a parallel GA by Ernesto Cipriani et al. [26]. Arbex [27] proposed an alternating objective genetic algorithm to efficiently solve the multi-objective public transport network design and frequency setting problem. The algorithm could overcome the problems of a large search space and multiple constraints. Ngamchai and Lovell [28] proposed a new model demonstrating how genetic algorithms can be manipulated to help optimize bus transit routing design, incorporating unique service frequency settings for each route. The model was applied on a benchmark network to test its efficiency, and performance results were presented. The results showed that the proposed model is more efficient than the binary-coded genetic algorithm benchmark. Based on solution methodologies for transit network planning and scheduling in the abovementioned studies, this paper further examines solution methodologies for the stop planning and timetables of CBs. In addition, the abovementioned studies have shown that genetic algorithms are highly efficient, accurate and scalable in models of CB stop planning and timetables. Therefore, the model proposed in this paper uses an improved immune genetic algorithm (IIGA).

The reviewed literature reveals that the existing network planning and scheduling approaches focus on conventional buses. Although a CB is part of an urban public transit system, it is different from a conventional bus. Thus, the abovementioned models and methods are not completely suitable for CBs. In modern large cities, the commuting distance is always long. The commuter hopes to enjoy the features of a high-quality bus travel service, such as on-time departure, no transfers, and on-time arrival. However, the complicated road conditions that occur during peak hours makes the traditional bus system consistently unreliable and unpunctual. CBs provide personalized, advanced and flexible demand-responsive transit service to commuters or other specific clientele. CBs constitute a demand-based transit system that aggregates the travel demand of individual passengers. CBs have fixed stops, lines, vehicles, timetables and prices; furthermore, CBs have the characteristics of serving one person, one direct stop and lane flexibility. CB stops include boarding stops in the boarding zone and alighting stops in the alighting zone. There are no intermediate stops. In addition, timetables of every stop in CBs are open to passengers. Based on large-scale travel demand, this paper proposes a model for the stop planning and timetables of CB. The model considers the comprehensive benefit of passengers, operators, and society. An IIGA is then designed to solve the model, and a real-world CB example based in Beijing is provided. The main improvements of the IIGA are multiple population design and transport operator design, memory library design, mutation probability design and crossover probability design, and the fitness calculation of the gene segment. Due to the characteristics of CBs, vehicle can use public transport lanes at peak times. The line in the middle area is not fixed, except for boarding stops and alighting stops, which must search for the shortest path based on traffic flow conditions. Therefore, this paper studies only CB stops in the boarding zone and alighting zone and does not use the driving line in the middle of the zone. This research provides an approach for public transport companies to establish CBs in metropolitan areas.

The remainder of this paper is organized as follows. The CB stop model and the formulation for stops and timetables of CBs are described in Section 2. Section 3 builds the model for the stop planning and timetables of CBs, and Section 4 solves the mode by improving the GA. The effectiveness of the IIGA is demonstrated by applying the algorithm to a real-world scenario in Section 5. Section 6 presents the conclusions of this research.

## 2 Formulation for the Stops and Timetables of CBs

CBs have fixed stops, lines, vehicles, timetable and prices and have the advantages of serving one person, one direct stop and lane flexibility. Because passenger travel demand is diverse, it is difficult to meet different passenger travel demands simultaneously. Hence, many factors must be considered in planning CB lines. There are three modes of CB stops:

(1) One bus on one line

**Fig 1:**
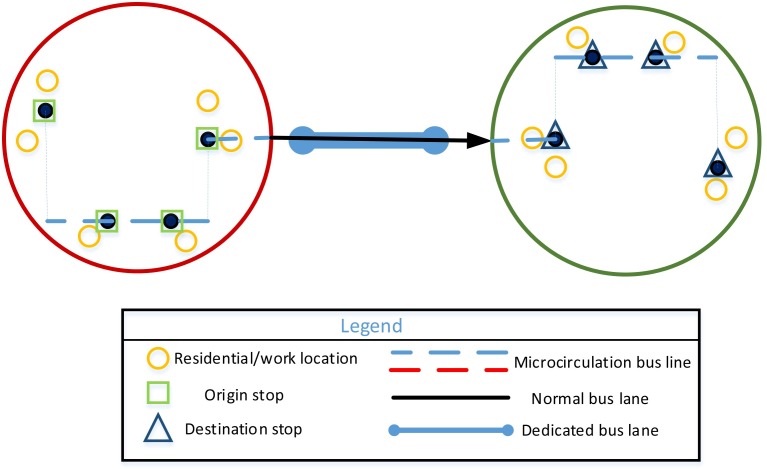
One customized bus on one line.

This model can meet passenger demand when passenger travel demand is small, as shown in Fig 1.

(2) Multiple buses on one line

**Fig 2:**
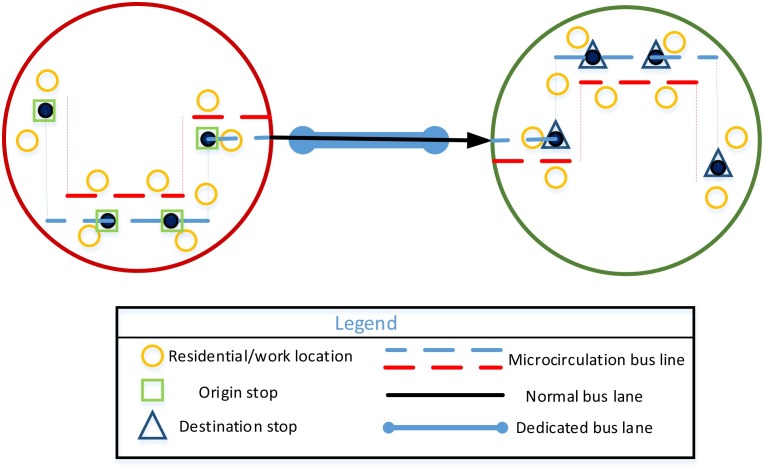
Multiple buses on one line.

The departure times of CB should be broader when the passenger travel demand is greater and the passenger travel time is more scattered. This model is shown in Fig 2.

(3) Multiple buses on multiple lines

**Fig 3:**
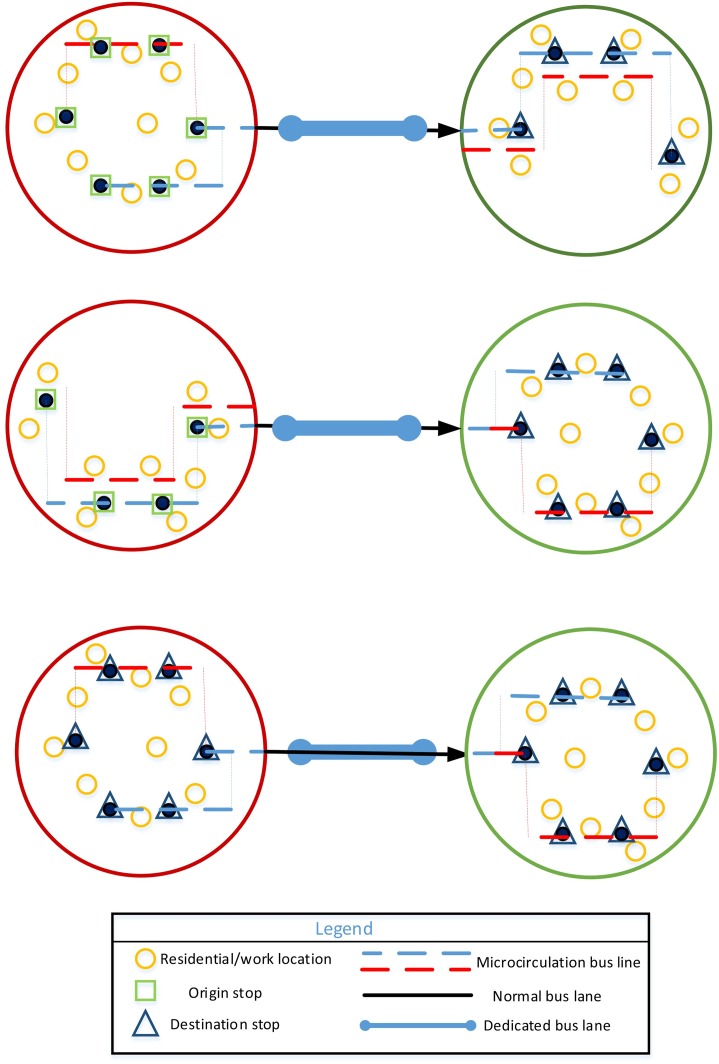
Multiple buses on multiple lines.

Additional lines should be opened when the CB network in a city is more mature, passenger demand is greater, and boarding and alighting stops are distributed in multiple zones. This model is shown in Fig 3.

CB lines can be represented by the following mathematical models. This paper assumes that a CB line consists of *λ*_1_ boarding stops and *λ*_2_ alighting stops. Boarding stops are represented by ${\text{s}}_{1},s_{2},\ldots ,s_{\lambda_{1}}$, and alighting stops are represented by $r_{1},r_{2},\ldots ,r_{\lambda_{2}}$. The vector lx represents all stops passed by each bus: $lx=\left\{s_{1},s_{2},\ldots s_{\lambda_{2}},r_{1},r_{2},\ldots r_{\lambda_{2}}\right\}$. The lines of customized buses between O and D are represented by the matrix *LX*, where m is the number of vehicles.

$$LX=\left[\begin{array}{c} lx^{1}\\ lx^{2}\\ \vdots \\ lx^{m} \end{array}\right]=\left[\begin{array}{cccccccc} s_{1}^{1} & s_{2}^{1} & \cdots & s_{\lambda_{1}}^{1} & r_{1}^{1} & r_{2}^{1} & \cdots & r_{\lambda_{2}}^{1}\\ s_{1}^{2} & s_{2}^{2} & \cdots & s_{\lambda_{1}}^{2} & r_{1}^{2} & r_{2}^{2} & \cdots & r_{\lambda_{2}}^{2}\\ \vdots & \vdots & \ddots & \vdots & \vdots & \vdots & \ddots & \vdots \\ s_{1}^{m} & s_{2}^{m} & \cdots & s_{\lambda_{1}}^{m} & r_{1}^{m} & r_{2}^{m} & \cdots & r_{\lambda_{2}}^{m} \end{array}\right]$$

If two boarding (alighting) stops are the same for the vehicle—namely,$s_{j}^{h}=s_{j+1}^{h}$ ($r_{j}^{h}=r_{j+1}^{h}$)—the vehicle parks at this stop once. Accordingly, the number of boarding (alighting) stops is ultimately less than *λ*_1_ (*λ*_2_). If all stops and ordering are the same for two vehicles, the two vehicles are in one line; only the departure times are different, as shown in Fig 2. If the solution is *m* = 1, only one vehicle participates in the operation, and *LX* is a vector this time, as shown in Fig 1.

According to the mathematical model of CB lines, the mathematical model of the relative stop timetable can also be represented by a matrix. The arrival time of all stops in the CB line can be expressed as $lt=\left\{t_{1}^{s},t_{2}^{s},\cdots ,t_{\lambda_{1}}^{s},t_{1}^{r},t_{2}^{r},\cdots ,t_{\lambda_{2}}^{r}\right\}$. The arrival time of all stops can also be represented by the following matrix for these vehicles:

$$LT=\left[\begin{array}{cccccccc} t_{1}^{s1} & t_{2}^{s1} & \cdots & t_{\lambda_{1}}^{s1} & t_{1}^{r1} & t_{2}^{r1} & \cdots & t_{\lambda_{2}}^{r1}\\ t_{1}^{s2} & t_{2}^{s2} & \cdots & t_{\lambda_{1}}^{s2} & t_{1}^{r2} & t_{2}^{r2} & \cdots & t_{\lambda_{2}}^{r2}\\ \vdots & \vdots & \ddots & \vdots & \vdots & \vdots & \ddots & \vdots \\ t_{1}^{sm} & t_{2}^{sm} & \cdots & t_{\lambda_{1}}^{sm} & t_{1}^{rm} & t_{2}^{rm} & \cdots & t_{\lambda_{2}}^{rm} \end{array}\right]$$

Among them, vehicle h between stops j+1 and j satisfies the following formulas:

$$t_{j+1}^{rh}-t_{j}^{rh}=\frac{l_{r_{j}^{h}r_{j+1}^{h}}}{v_{avg}}+T\left(r_{j}^{h}\right)$$ (2-1)

$$t_{j+1}^{sh}-t_{j}^{sh}=\frac{l_{s_{j}^{h}s_{j+1}^{h}}}{v_{avg}}+T\left(s_{j}^{h}\right)$$ (2-2)

$$t_{1}^{rh}-t_{\lambda_{1}}^{sh}=\frac{l_{s_{\lambda_{1}}^{h}r_{1}^{h}}}{v_{avg}}+T\left(s_{\lambda_{1}}^{h}\right)$$ (2-3)

where *v*_*avg*_ is the CB travel speed and *T*(*x*) is the CB dwell time at stop x.

All stop dwell times *T*(*x*) of vehicle h are related to the number of boarding and alighting stops as follows:

$$T\left(r_{j}^{h}\right)=\sum_{i=1}^{n}\delta_{j}\left(i,h\right)YN\left(i,h\right)t_{s}+t_{g}$$ (2-4)

$$T\left(s_{j}^{h}\right)=\sum_{i=1}^{n}\beta_{j}\left(i,h\right)YN\left(i,h\right)t_{x}+t_{g}$$ (2-5)

where *t*_*s*_ is each passenger’s boarding time, *t*_*g*_ is the necessary vehicle dwelling time, *t*_*x*_ is each passenger’s alighting time, *YN*(*i*, *h*) represents whether passenger i takes the CB h, *δ*_*j*_(*i*, *h*)*YN*(*i*, *h*) represents whether passenger i boards at stop j of vehicle h, and *β_j_*(*i*,*h*)*YN*(*i*,*h*) represents whether passenger i alights at stop j of vehicle h. Eq 3-1 is used to calculate *YN*(*i*, *h*).

According to the abovementioned formulas, the CB stop timetable can be accurately calculated if the departure time, travel path and number of boarding stops are known.

## 3 Model for the Stop Planning and Timetables of CBs

This paper builds a model for the stop planning and timetables of CBs considering the passenger, operator, and societal perspectives based on passenger travel space and time demand.

### 3.1 Objective function

CB operation must consider the passenger, operator, and societal perspectives. Passengers seek to minimize their commuting time and maximize their scheduling time, operators seek to maximum operational income while minimizing operational costs, and societal benefits should be considered in selecting the number of operating CBs.

The objective function is calculated according to the three aspects of passengers, operators, and societal benefits.

#### 3.1.1 Passengers’ perspective

The time cost of passengers consists of in-vehicle cost, waiting cost, and arrival time cost ahead of schedule. When the time cost of passengers is calculated, one should first consider whether passengers choose CBs.

(1) Whether vehicle h meets the travel demand of passenger i

There are three main standards used to determine whether vehicle h meets the travel demand of passenger i: (1) the boarding and alighting stops in the line for passenger i; (2) whether the actual vehicle arrival time is earlier than the passenger’s expected time of arrival; and (3) passengers who screen stops will prefer to choose vehicle h when some vehicles pass a stop. The condition for meeting the first standard is $x_{i}^{s},y_{i}^{r}\in lx^{h}$. To meet the second standard, one must first determine whether the passenger’s alighting stop is one of the stops. If $r_{j}^{h}-y_{i}^{r}=0\;(j=1,2,\cdots ,\lambda_{2})$, passenger i alights at stop j of vehicle h. If $t_{i}^{sd}\geq t_{j}^{rh}$—namely, the passenger’s expected latest time of arrival is not earlier than the passenger alighting time at stop j of vehicle h—passenger i can take vehicle h; otherwise, passenger i cannot take vehicle h. If $t_{i}^{sd}\gg t_{j}^{rh}$—namely, the difference between $t_{i}^{sd}$ and $t_{j}^{rh}$ is greater than critical value T—the passenger does not take vehicle h because the wasted time is excessively high for the passenger. To meet the last standard, passengers are considered to give preference to the vehicle whose actual starting time is latest. If two or more vehicles can meet the passenger travel demand, passengers choose the vehicle for which $t_{j}^{rh}$ is maximum.

Whether passenger i takes the CB h is described as follows:

YN(i,h)=δ(i,h)β(i,h)τ(i,h)θ(i,h) (3-1)

where *δ*(*i*, *h*) indicates whether passenger i boards at the boarding stop of vehicle h and $\delta \left(i,h\right)=\sum_{j=1}^{\lambda_{1}}\delta_{j}\left(i,h\right)$.

*δ*_*j*_(*i*, *h*) indicates whether passenger i boards at boarding stop j of vehicle h and $\delta_{j}\left(i,h\right)=\{\begin{array}{c} 1\;,\;s_{j}^{h}-x_{i}=0\\ 0\;,\;s_{j}^{h}-x_{i}\neq 0 \end{array}$.

*β*(*i*, *h*) indicates whether passenger i alights at alighting stop j of vehicle h and $\beta \left(i,h\right)=\sum_{j=1}^{\lambda_{2}}\beta_{j}\left(i,h\right)$.

*β*_*j*_(*i*, *h*) indicates whether passenger i alights at boarding stop j of vehicle h and $\beta_{j}\left(i,h\right)=\{\begin{array}{c} 1\;,\;r_{j}^{h}-y_{i}=0\\ 0\;,\;r_{j}^{h}-y_{i}\neq 0 \end{array}$.

*τ*(*i*, *h*) indicates whether the actual arrival time vehicle h is earlier than the expected time of passenger i. If $r_{j}^{h}-y_{i}=0$, then $\tau \left(i,h\right)=\{\begin{array}{cc} 1 & 0\leq t_{i}^{sd}-t_{j}^{rh}\leq T\\ 0 & t_{i}^{sd}-t_{j}^{rh}>T\text{ or }t_{i}^{sd}-t_{j}^{rh}<0 \end{array}$. If $r_{j}^{h}-y_{i}\neq 0$, then *τ*(*i*, *h*) = 0.

*θ*(*i*, *h*) indicates whether the starting time of vehicle h is the latest time at which passenger i can take the vehicle. If $\sum_{h=1}^{m}\delta \left(i,h\right)\beta \left(i,h\right)\tau \left(i,h\right)\geq 2$, then passenger i can choose to take CB from at least two vehicles; at this time, passenger i must choose a late departure time. If *δ*(*i*, *h*) *β*(*i*, *h*) *τ*(*i*, *h*) = 1 and $s_{j}^{h}-x_{i}=0\;(h=1,2,3,\ldots ,m;\;j=1,2,3,\ldots ,\lambda_{1})$, then $t_{j}^{sh}$ is subordinated to set *H*. If $t_{j}^{sh}=\text{max }H$ and $t_{j}^{sh}\in H$, then *θ*(*i*, *h*) = 1; otherwise, *θ*(*i*, *h*) = 0.

(2) Time cost of passengers

The time cost of passengers is divided into three parts. The time cost of passengers is represented by *C*_*t*_. First, this paper assumes that the passenger waiting time is zero. Second, the time wasted when a passenger’s actual arrival time is earlier than the passenger’s expected arrival time is represented by *C*_*d*_(*i*). Finally, travel cost is represented by *C*_*l*_(*i*). The travel time cost of passenger i in travel is calculated as follows:

$$C_{t}\left(i\right)=C_{d}\left(i\right)+C_{l}\left(i\right)$$ (3-2)

The wasted time is calculated as follows:

$$C_{d}\left(i\right)=\sum_{h=1}^{m}\sum_{j=1}^{\lambda_{2}}f_{i}\left(t_{i}^{sd}-t_{j}^{rh}\right)\beta_{j}\left(i,h\right)YN\left(i,h\right)$$ (3-3)

The traveling cost is calculated as follows:

$$C_{l}\left(i\right)=\sum_{h=1}^{m}\sum_{k=1}^{\lambda_{2}}\sum_{j=1}^{\lambda_{2}}g_{i}\left(t_{j}^{rh}-t_{k}^{sh}\right)\delta_{k}\left(i,h\right)\beta_{j}\left(i,h\right)YN\left(i,h\right)$$ (3-4)

In this paper, the time value is calculated by the income approach, which reflects that the wasted time of passengers during travel leads to reduced income because the passengers are unable to work during that time. The calculation method is as follows:

$$\omega_{t}\left(i\right)=NC\left(i\right)/NT\left(i\right)$$ (3-5)

where *NC*(*i*) is the personal annual income of passenger i and *NT*(*i*) is the annual working time of passenger i.

The income approach reflects the difference in time valuation between different passengers. For the income inequality of passengers in the case of the same price, travel time savings is more important for a higher passenger revenue. Furthermore, the travel time value is proportional to passenger income as follows:

$$g_{i}\left(x\right)=\alpha_{g}\left(i\right)\omega_{t}\left(i\right)x$$ (3-6)

where *α*_*g*_(*i*) is the loss factor of the travel time of passenger i.

For any type of traffic, the CB can arrive at the destination in advance. The wasted time value when the CB arrives at the destination in advance is proportional to passenger income as follows:

$$f_{i}\left(x\right)=\alpha_{f}\left(i\right)\omega_{t}\left(i\right)x$$ (3-7)

where *α*_*f*_(*i*) is the loss factor of wasted time for passenger i when the CB arrives at the destination ahead of schedule.

For the loss factor of time, it is higher when passenger i considers that the period is no value. Thus, *α*_*g*_(*i*) is typically greater than *α*_*f*_(*i*).

The passenger’s total time cost is described as follows:

$$C_{t}=\sum_{i=1}^{n}C_{t}\left(i\right)$$ (3-8)

(3) Passenger travel value

Commuter travel is aimed at going to work, creating societal value, and obtaining a certain salary. Thus, passenger travel also has a certain value for passengers. Passenger travel value is represented by R_x_.

This paper assumes that passenger travel value and travel time are related to wages as follows:

$$R_{x}\left(i\right)=\alpha_{x}\left(i\right)\omega_{t}\left(i\right)\Delta t\left(i\right)$$ (3-9)

where *α*_*x*_(*i*) is the proportion of passenger travel value and the wages per unit time and Δ*t*(*i*) is the travel time of passenger i.

CB passenger travel value is described as follows:

$${R^{\prime }}_{x}\left(i\right)=\sum_{h=1}^{m}\sum_{k=1}^{\lambda_{2}}\sum_{j=1}^{\lambda_{1}}\alpha_{x}\left(i\right)\omega \left(i\right)\delta_{k}\left(i,h\right)\beta_{j}\left(i,h\right)YN\left(i,h\right)\left(t_{j}^{rh}-t_{k}^{sh}\right)$$ (3-10)

Thus, all CB passenger travel values are described as follows:

$$R_{x}=\sum_{i=1}^{n}{R^{\prime }}_{x}\left(i\right)$$ (3-11)

#### 3.1.2 Operators’ perspective

From the operators’ perspective, this paper considers that operating a CB consists mainly of operational income and operational cost.

(1) Operational income

The operational income of a CB is derived from the fare income of the CB (other income can be neglected). The CB fare is based on the operating distance, which is an integer. The fare income is calculated as follows:

$$R_{s}=\sum_{h=1}^{m}\sum_{i=1}^{n}\left\lceil L_{x_{i}^{s}y_{i}^{r}}\cdot \omega_{p}+P_{g}\right\rceil YN\left(i,h\right)$$ (3-12)

where *R*_*s*_ is societal benefits, *ω*_*p*_ is the fare per unit travel in kilometers and *P*_*g*_ is the starting fare for the CB.

(2) Operational cost

The operational cost of a CB refers to all expenses in the form of money associated with the consumption of passenger travel, which consists of driver wages and welfare, vehicle fuel and depreciation fees in this paper. The operational cost is calculated as follows:

$$C_{y}=C_{pd}+C_{yr}+C_{cz}$$ (3-13)

where *C*_*y*_ is the operational cost, *C*_*pd*_ is the driver cost, *C*_*yr*_ is the fuel cost, and *C*_*cz*_ is the depreciation cost.

The driver cost consists of fixed wages and variable wages. Variable wages are related to travel kilometers. The driver cost is calculated as follows:

$$C_{pd}=mC_{pdg}+\sum_{h=1}^{m}\left(t_{\lambda_{2}}^{rh}-t_{1}^{sh}+t_{cc}^{h}+t_{cd}^{h}\right)v_{avg}\omega_{d}$$ (3-14)

where *C*_*pdg*_ is the driver’s fixed cost, $t_{cc}^{h}$ is the time required to drive vehicle h from the parking lot to the first stop, $t_{cd}^{h}$ is the time required to drive vehicle h from the end of the transport mission back to the parking lot, and *ω*_*d*_ is the unit time cost of driving the vehicle.

Fuel cost is proportional to driving distance as follows:

$$C_{yr}=\sum_{h=1}^{m}(\sum_{i=1}^{\lambda_{1}-1}L_{s_{i}^{h}s_{i+1}^{h}}+\sum_{i=1}^{\lambda_{2}-1}L_{r_{i}^{h}r_{i+1}^{h}}+L_{s_{\lambda_{1}}^{h}r_{1}^{h}}+L_{cc}^{h}+L_{cd}^{h})\omega_{r}$$ (3-15)

where *ω*_*r*_ is the fuel cost per unit distance, $L_{cc}^{h}$ is the driving distance of vehicle h from the parking lot to the first stop, and $L_{cd}^{h}$ is the driving distance of vehicle h from the end of the transport mission back to the parking lot.

The value of the vehicle will gradually decrease because of wear and tear. This decrease in value is the depreciation cost, which is considered the average vehicle value of fixed assets according to the service life of the vehicle in this paper. The depreciation cost for each operation of the vehicle is calculated as follows:

$$C_{cz}=\overset{m}{\underset{h=1}{\sum }}C_{g}^{h}/365\varepsilon^{h}n_{c}^{h}$$ (3-16)

where $C_{g}^{h}$ is the purchase cost of vehicle h, *ε*^*h*^ is the service life of vehicle h, and nch is the number of times that vehicle h is used in one day.

#### 3.1.3 Societal perspective

This paper considers that the societal benefits mainly include road congestion and pollutant emissions. Because the CB service object contains private commuters, if the passengers do not take the CB, then the commuter who drives a private car will increase road congestion and increase pollution gas emissions.

(1) Reduction in traffic congestion cost

The congestion cost is proportional to the road area and the driving distance as follows:

$$C_{yj}=\omega_{yj}S_{zd}L_{zx}$$ (3-17)

where *ω*_*yj*_ is the congestion cost per unit area, *S*_*zd*_ is the occupied road area of vehicles, and *L*_*zx*_ is the vehicle driving distance.

Thus, for a certain number of passengers, choosing to take a CB will reduce congestion costs relative to choosing private cars as follows:

$$C_{yj}^{\prime }=\omega_{yj}\sum_{h=1}^{m}\left(\sum_{i=1}^{n}S_{zd}^{car}L_{x_{i}^{s}y_{i}^{r}}YN\left(i,h\right)-S_{zd}^{bus}(\sum_{i=1}^{\lambda_{1}-1}L_{s_{i}^{h}s_{i+1}^{h}}+\sum_{i=1}^{\lambda_{2}-1}L_{r_{i}^{h}r_{i+1}^{h}}+L_{s_{\lambda_{1}}^{h}r_{1}^{h}}+L_{cc}^{h}+L_{cd}^{h})\right)$$ (3-18)

where $S_{zd}^{car}$ is the occupied road area of cars and $S_{zd}^{bus}$ is the occupied road area of a CB.

(2) Reduction in environmental pollution cost

For a certain number of passengers, choosing to take a CB will reduce the environmental pollutant cost relative to choosing private cars as follows:

$$C_{wb}^{\prime }=\sum_{h=1}^{m}\left(\sum_{i=1}^{n}\omega_{wb}^{car}L_{x_{i}^{s}y_{i}^{r}}YN\left(i,h\right)-\omega_{wb}^{bus}(\sum_{i=1}^{\lambda_{1}-1}L_{s_{i}^{h}s_{i+1}^{h}}+\sum_{i=1}^{\lambda_{2}-1}L_{r_{i}^{h}r_{i+1}^{h}}+L_{s_{\lambda_{1}}^{h}r_{1}^{h}}+L_{cc}^{h}+L_{cd}^{h})\right)$$ (3-19)

where $\omega_{wb}^{car}$ is the external unit cost of the discharged pollutant per kilometer traveled by car and $\omega_{wb}^{bus}$ is the external unit cost of the discharged pollutant per kilometer traveled by CB.

In summary, societal benefits include the reduction in traffic congestion and environmental pollution costs as follows:

$$R_{w}=C_{yj}^{\prime }+C_{wb}^{\prime }$$ (3-20)

where *R*_*w*_ is the operational income.

### 3.2 Objective function, constraints and assumptions

CB stop design must satisfy constraints at the service level, such as the number of stops, as well as constraints on the lines and stop mileage and the requirements for the vehicle load factor. In addition, the stop design should ensure that passengers take the same CB when they are at the same starting stop.

Based on the foregoing considerations, the objective function of the model is as follows:

$$\text{Max }Z=-C_{t}+R_{x}+R_{w}-C_{y}+R_{s}$$ (3-21)

subject to

$$\lambda_{\text{3}}\leq \sum_{i=1}^{n}YN(i,h)\leq \lambda_{4}\;(h=1,2,\ldots ,m)$$ (3-22)

$$\lambda_{5}\leq L_{s_{\lambda_{1}}^{h}r_{1}^{h}}\leq \lambda_{6}$$ (3-23)

$$L_{s_{i}^{h}s_{i+1}^{h}}\leq \lambda_{7}\;(i=1,2,..,\lambda_{1});\;L_{r_{i}^{h}r_{i+1}^{h}}\leq \lambda_{8}\;(i=1,2,..,\lambda_{2})$$ (3-24)

$$\sum_{i=1}^{\lambda_{1}}L_{s_{i}^{h}s_{i+1}^{h}}\leq \lambda_{9},\;\sum_{i=1}^{\lambda_{2}}L_{r_{i}^{h}r_{i+1}^{h}}\leq \lambda_{10}$$ (3-25)

$$\sum_{h=1}^{m}\varphi_{k}^{sh}\leq \lambda_{11},\;\sum_{h=1}^{m}\varphi_{k}^{rh}\leq \lambda_{12}\;(k=1,2,\ldots ,K)$$ (3-26)

Constraint (3-22): The CB will operate when the number of passengers is at least *λ*_3_ on a line, and the rated passenger capacity of the CB is *λ*_4_.

Constraint (3-23): The upper bound *λ*_6_ indicates that the CB line should not be excessively long, which avoids the problem of long travel time. The lower bound *λ*_5_ indicates that the CB line should not be extremely short. When the travel path is sufficiently short, the original public transport system can also meet passenger demands, and the high CB fare reduces the competitiveness of this mode of transportation.

Constraint (3-24): *λ*_8_(*λ*_9_) represents the longest distance between boarding stops (alighting stops).

Constraint (3-25): *λ*_9_(*λ*_10_) represents the longest line length between boarding stops (alighting stops).

Constraint (3-26): *λ*_11_(*λ*_12_) represents the maximum number of vehicles that can pass through the same boarding stop (alighting stop), where $\varphi_{k}^{sh}$ indicates whether stop k is a boarding stop of vehicle h and $\varphi_{k}^{sh}=\left\{\begin{array}{l} 1,s_{j}^{h}-k=0(1,2,\ldots ,9)\\ 0,s_{j}^{h}-k\neq 0(1,2,\ldots ,9) \end{array}\right\}$, $\varphi_{k}^{rh}$ indicates whether stop k is an alighting stop of vehicle h and $\varphi_{k}^{rh}=\left\{\begin{array}{l} 1,r_{j}^{h}-k=0(1,2,\ldots ,9)\\ 0,r_{j}^{h}-k\neq 0(1,2,\ldots ,9) \end{array}\right\}$, and K is the total number of numbered stops.

The model makes the following assumptions:

1. The CB driving speed is fixed.
2. The phenomenon of early or late arrival for passengers and vehicles is nonexistent.
3. Traffic area roads are networked.
4. The required boarding and alighting times are fixed for each passenger.
5. Passenger demand is not canceled.

## 4 Solution Method

The genetic algorithm (GA) is based on the concept of natural selection. A GA searches for the optimal solution by simulating the natural process of evolution and is one of the most commonly used artificial intelligence algorithms. GAs have been successfully applied in many fields due to their simplicity, robustness and adaptability to parallel distributed processing. However, Belew R K [29] believed GAs still have certain shortcomings, such as premature convergence and lack of local search ability. The immune genetic algorithm (IGA) is a new type of combined optimization method that incorporates the characteristics of the biological immune system based on a GA. An IGA has high antigen recognition, self-regulation and other functions based on the robustness of the GA, which performs well in terms of search speed, global search capability and local search capability. Jiao L and Wang L [30] verified that the IGA is feasible and effective and is conducive to alleviating the degeneration phenomenon in the original GA based on examples of the traveling salesman problem (TSP). The variables are too numerous and difficult to obtain for the model proposed in Section 3; thus, this paper solves the model for the stop planning and timetables of CBs using a heuristic algorithm with an IGA, which searches the feasible solution space rapidly and exhibits an excellent global searching ability. Because the IGA is a single-population design, its global search ability is poor, and it easily falls into a local optimal solution. The fixed crossover operator and mutation operator can prevent the algorithm from being adjusted according to the actual situation of the group and from reflecting the different requirements of the population’s antibodies in different evolutionary stages, thus resulting in a poor optimized effect. This paper proposes an IIGA according to the characteristics of the formulated model. The main improvements are as follows:

1. Multiple-population design. The introduction of multiple-population design can enrich the evolutionary direction of different populations and introduce the superior antibody of each subpopulation using the migration operator, which can effectively prevent the premature convergence phenomenon.
2. Memory library design. The introduction of memory library design can ensure that better antibody can be inherited by the next generation, preventing the antibody degradation phenomenon.
3. Designing adaptive crossover probability and mutation probability. The evolution of the crossover probability and that of the mutation probability are determined according to the diversity and excellence of the previous population generation, which prevents the premature convergence phenomenon.
4. Fitness calculation of the gene segment. Because the model solution is composed of a number of stops and the first stop time, it should be as far as possible to reduce the crossover and mutation operation for a vehicle of excellent performance; thus, the convergence to the optimal solution will be accelerated.

### 4.1 Designing the IIGA

(1) Antibody coding

**Fig 4:**
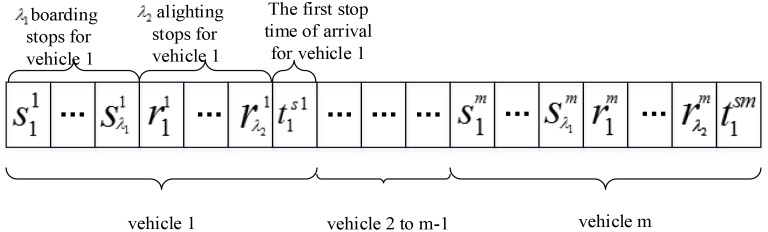
Structure of the antibodies.

The model decision variables are *LX* and all vehicles departure times $t_{1}^{sh}$. Because the number of decision variables is greater, a binary code cannot be adopted; thus, this paper adopts real code, and the length of the antibody is the number of decision variables. For each vehicle, all stops *lx*^*h*^ and starting time $t_{1}^{sh}$ are taken as a unit to form an antibody according to the order of the vehicles, as shown in Fig 4.

(2) Initial antibody population generation

The initial antibody population size M and the length of the antibody in population (*λ*_1_ + *λ*_2_ + 1)*m*, *m* ∈ [1, *m*_max_] are set. Each antibody randomly selects a different length structure—namely, it randomly selects the value of m; then, the population contains a number of different vehicles.

The specific methods for determining the internal variables of the antibody are as follows: First, all passengers with a given boarding stop demand and alighting stop demand are placed into boarding stop set *SC* and alighting stop set *XC*, respectively. Second, $s_{1}^{h}$ is randomly selected from set *SC*, $s_{2}^{h}$ selects the stop number that is closest to $s_{1}^{h}$ in set *SC*, $s_{3}^{h}$ selects the stop number that is closest to $s_{2}^{h}$ in set *SC*, etc. In addition, $r_{1}^{h}$ is randomly selected from set *XC*, $r_{2}^{h}$ selects the stop number that is closest to $r_{1}^{h}$ in set *XC*, $r_{3}^{h}$ selects the stop number that is closest to $r_{2}^{h}$ in set *XC*, etc. $t_{1}^{sh}$ is randomly generated from a range of values. The initial population attempts to set the stops nearby because the stop number rule is designed according to the ascending sequential order of the X- and Y-coordinates of the stops.

In this paper, multiple-population design divides the antibody population into three subgroup antibody populations—namely, an advanced antibody population, a behindhand antibody population and an intermediate antibody population. Under the condition that the crossover probability and mutation probability have been obtained, the different subgroup antibody populations must be multiplied by different correction values, which increases the crossover probability and mutation probability and reduces the crossover probability and mutation probability of the behindhand antibody population. The correction values of the advanced, behindhand, and intermediate antibody populations are *γ*_5_, *γ*_6_ and *γ*_7_, respectively. The process involves the independent calculation of each antibody affinity and immune genetic operation. The subgroup antibody population will exchange several optimal antibodies every few evolutionary algebraic operations; in this manner, a more excellent antibody is introduced at the same time and enriches the diversity of the antibody population, which prevents immature and early convergence.

(3) Affinity between antibodies and antigens

The affinity between antibodies and antigens can be obtained by changing the objective function and constraints: A more optimized antibody is associated with a greater objective function value of the corresponding objective function and greater affinity. The calculated formula is as follows:

$$A_{v}=Z(v)+\sum_{n=1}^{I}pc\times \eta_{n}(v)$$ (4-1)

where *pc* is a penalty constant, *η*_*n*_ (*v*) = {0,1} indicates whether antibody V violates the nth constraint, and *I* is the number of constraints.

Thus, this paper proposes the concept of gene segments. Because the solution of the model is composed of the stops of all vehicles and the first stop time, the solution should minimize the crossover and mutation operations for a vehicle of the excellent performance and thereby speed up the convergence of the optimal solution. A vehicle in an antibody can be viewed as a gene segment. The fitness calculation of the gene segment is described as follows:

$$f_{vh}=Z(v_{h})+\sum_{n=1}^{I}pc\times \lambda_{n}(v_{h})$$ (4-2)

where *v*_*h*_ is the gene values, which are fixed from (*λ*_1_+*λ*_2_+1)(*h*−1)+1 to (*λ*_1_+*λ*_2_+1)*h* and are 0 otherwise.

(4) Transport operator

In this paper, the design concept of the transport operator is as follows: The algorithm defines transport interval *γ*_8_ and transport quantity *γ*_9_, where *γ*_8_ is the frequency of transport behavior between subgroup antibody populations and *γ*_9_ is the number of each transport antibody. When the IGA evolves to *γ*_8_ over multiple generations, the best *γ*_9_ antibodies for each subgroup antibody population are transferred to other antibody groups.

(5) Memory library design

The memory library design can prevent the destruction of better antibodies by a crossover operator or mutation operator and retain better antibodies in the subsequent generation to accelerate the algorithm convergence. The memory library design is performed as follows:

1. Setting the initial memory library. The memory library size is J; the previous J antibodies are placed into the initial memory library of each subspecies population in descending order of the subpopulation antibody affinity.
2. Updating the memory library. After genetic manipulation, the affinity of the subpopulation antibody is sorted. If the affinity of the newly generated antibody is higher than that of antibodies in the original memory library, these new antibodies will be placed into the memory library; then, the same number of antibodies for which the affinity is the worst in the memory bank will be replaced. Otherwise, the memory library remains the same. The same antibody is not selected to ensure the diversity of the memory library.
3. The antibodies of the memory library in the next generation. After each memory library is updated, the antibodies in the memory library are directly inherited by the next generation.

(6) Antibody of promotion and inhibition

In the iterative process of the IGA, the antibody concentration is increased to a certain value that is inhibited, improving the production and selection probability of the antibody for which the concentration is lower. The affinity between antibodies is calculated to determine the concentration of the antibody and thus determine whether the antibody is inhibited or promoted.

1) Calculation of the affinity between antibodies

**Fig 5:**
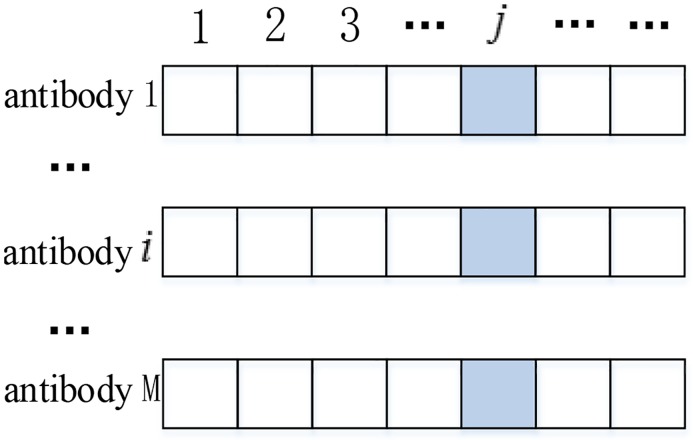
Shannon information entropy of each gene.

Lee W, Lee S, Lee B H, et al. [31] proposed that the affinity between antibodies is calculated based on Shannon information entropy theory. First, the degree of similarity between the antibodies is calculated. Each antibody corresponds to a solution *X*_*i*_. The affinity degree *A*_*ij*_ between antibody *X*_*i*_ and antibody *X*_*j*_ indicates the degree of similarity between the antibodies. The Shannon information entropy is shown in Fig 5.

The information entropy of the gene at location j is as follows:

$$H_{j}\left(M\right)=\sum_{i}-p_{ij}\text{ln}p_{ij}$$ (4-3)

where *p*_*ij*_ is the appearance probability of the j*th* position in the antibody for allelic gene i.

The diversity of the average information entropy is as follows:

$$H\left(M\right)=\overset{(\lambda_{1}+\lambda_{2}+1)m_{\text{max}}}{\underset{j=1}{\sum }}H_{j}\left(M\right)/\left((\lambda_{1}+\lambda_{2}+1)m_{\text{max}}\right)$$ (4-4)

According to the definition of entropy, the affinity between the antibodies is as follows:

$$A_{ij}=1/\left(1\text{+}H\left(2\right)\right)$$ (4-5)

According to the affinity between antibodies, the antibody concentration can be calculated as follows:

$$\rho_{i}=\underset{j}{\sum }S_{ij}/M$$ (4-6)

where $S_{ij}=\{\begin{array}{c} 1\;,A_{ij}\geq T_{acl}\;\\ 0\;,A_{ij}<T_{acl}\; \end{array}$ and *T*_*acl*_ is the set threshold for antibody affinity (0.9≤*T*_*acl*_≤1).

2) Antibody viability

Viability is the ability to survive to the next iteration of the antibody and is calculated as follows:

$$e_{v}=A_{v}/\rho_{v}$$ (4-7)

where *ρ*_*v*_ is the antibody concentration between the antibody and antibody v.

(7) Selection operator

In this paper, the operation selection method is a roulette wheel. The probability of antibodies being selected is based on the antibody viability *e*_*v*_. The roulette wheel method operates as follows: A random number r is randomly generated between 0 and 1 and compared with the cumulative probability *P*_*v*_ of each antibody viability. If *P*_*v*−1_<*r*≤*P*_*v*_, antibody v will be inherited by the next generation.

The cumulative probability of antibody v is as follows:

$$P_{v}=\overset{v}{\underset{t=1}{\sum }}e/\overset{M}{\underset{t=1}{\sum }}e_{t}$$ (4-8)

According to the roulette wheel selection, the probability *p*_*v*_ of the antibody is selected as follows:

$$P_{v}=e_{v}/\overset{M}{\underset{t=1}{\sum }}e_{t}$$ (4-9)

(8) Adaptive crossover operator based on the gene segment

**Fig 6:**
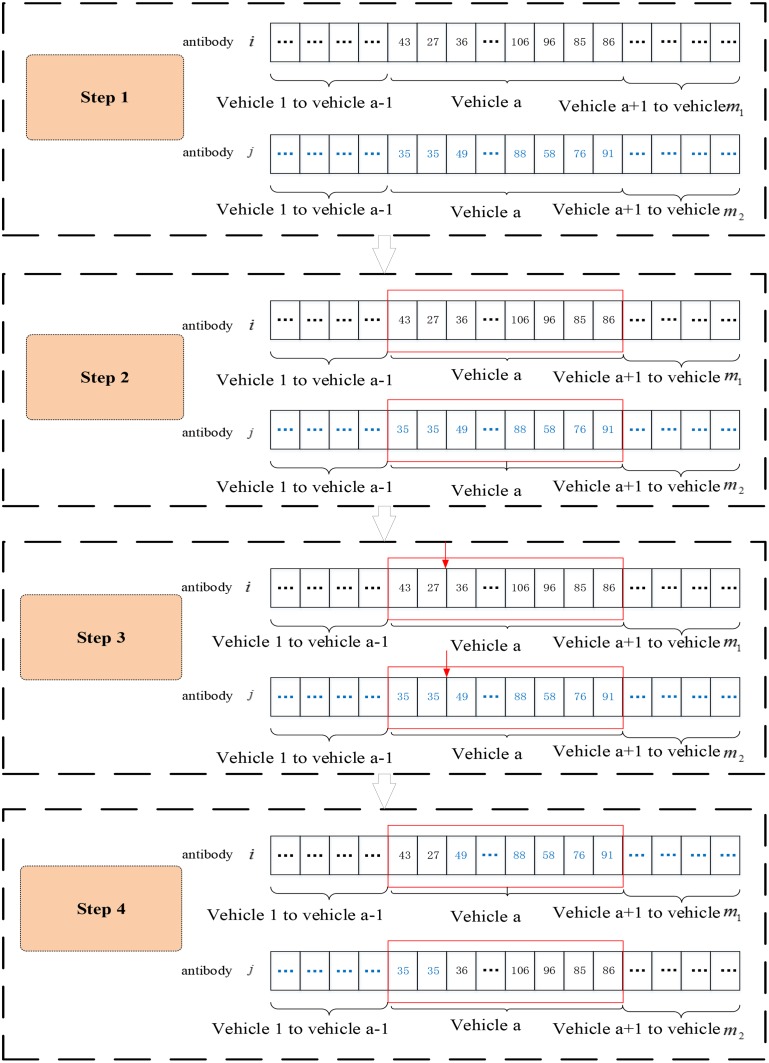
Crossover of the IIGA.

In this paper, an improved single-point crossover operator can increase the crossover probability of the diversity of antibodies and reduce the crossover operation of the gene segments with excellent performance. The steps of this operator are shown in Fig 6.

Step 1. Select two antibodies to cross: Determine whether the two antibodies cross based on the crossover probability. The probability of antibody crossover is calculated by an adaptive method and is determined by the antibody population diversity of the current generation. According to the expression of the logistic curve equation, the crossover probability is defined as follows:

$$P_{c}=k_{1}/\left(1+\text{exp}\left(1/(1+H(M))\right)\right)$$ (4-10)

Step 2. Select the gene segment in the crossover location: When selecting the shorter antibody from two crossover antibodies, the probability that each segment is selected depends on the fitness of the gene segment. The probability of segment h is selected as follows:

$${\text{P}}_{vh}=\left(1/f_{vh}\right)/\overset{m}{\underset{h=1}{\sum }}1/f_{vh}$$ (4-11)

Step 3. Randomly select the crossover position in the gene segment.

Step 4. Determine the antibody sequence after exchanging each crossover point.

(9) Adaptive mutation operator based on the gene segment

**Fig 7:**
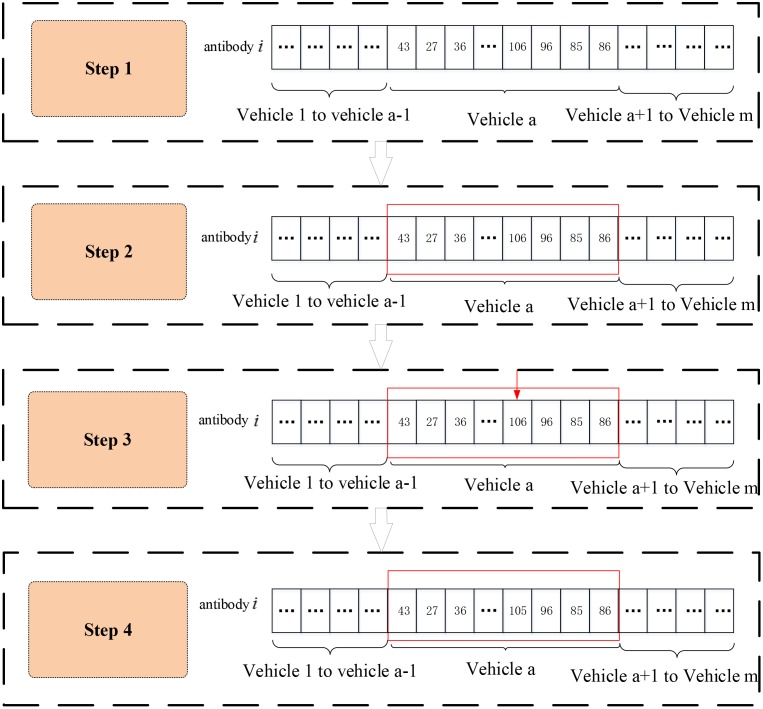
Mutation of the IIGA.

The mutation operator design resembles the crossover operator, reducing the crossover probability of the antibody diversity and reducing the crossover operation of the excellent gene segment. The corresponding steps are shown in Fig 7.

Step 1. Select two antibodies to implement the mutation: The antibody mutation probability, which is calculated by an adaptive approach and is determined by the antibody population current diversity, is used to determine whether mutation is needed.

The mutation probability is defined as follows:

$$P_{m}=k_{2}/\left(1+\text{exp}\left(H(M)\right)\right)$$ (4-12)

Step 2. Select a gene segment in the mutation location: The probability that the segment is selected depends on the fitness of the gene segment. The probability that segment h is selected is as follows:

$$P_{vh}=\left(1/f_{vh}\right)/\overset{m}{\underset{h=1}{\sum }}1/f_{vh}$$ (4-13)

Step 3. Randomly select the mutation position in the gene segment.

Step 4. Change the gene value of the mutation point.

(10) Immune operator

The immune operator is divided into three main parts: the extraction vaccine, vaccination and immune selection. In this paper, the extraction vaccine is based on the passenger travel demand, with *γ*_1_ typical boarding stops, *γ*_2_ typical alighting stops, and *γ*_3_ dense departure times. The method for selecting typical boarding stops and typical alighting stops is as follows: *γ*_1_ typical boarding stops and *γ*_2_ typical alighting stops for which the demand is maximum are selected after summarizing and analyzing the passenger travel demand of boarding stops and alighting stops. The dense departure times are selected as follows: The travel time is estimated according to the distance between the boarding zone and alighting zone, then a detour coefficient *α*_*rx*_ is set and detour travel time is *α*_*rx*_ times as long as the distance of the travel time.*γ*_3_ expected arrival times are then chosen, which causes all passengers to require as much as possible in the range of *γ*_4_ minutes before and after expected arrival times. *γ*_3_ dense departure times are calculated based on the detour travel time.

**Fig 8:**
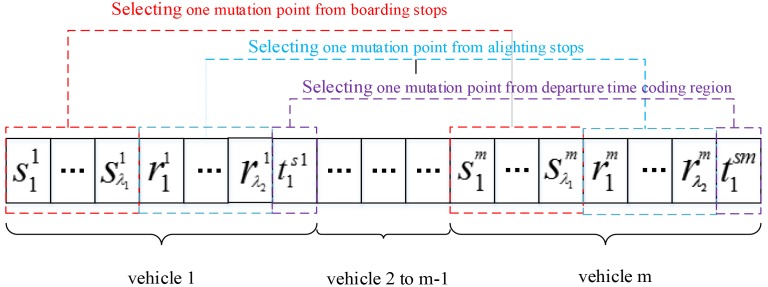
Immunity process.

The vaccination method is performed as follows: The gene locus of the required vaccination antibody is divided into three parts. The first part is the boarding stop coding region—namely, the range of [(*λ*_1_ *+ λ*_2_ + 1)*h* + 1, (*λ*_1_ *+ λ*_2_ + 1)*h* + *λ*_1_] (*h* = 0,1,..,*m*_max_ − 1) for the antibody. The second part is the alighting stop coding region—namely, the range of [(*λ*_1_ *+ λ*_2_ + 1)*h* + *λ*_1_ + 1, (*λ*_1_ *+ λ*_2_ + 1)*h* + *λ*_1_+*λ*_2_] (*h* = 0,1,..,*m*_max_ − 1) for the antibody. The third part is the departure time coding region—namely, {(*λ*_1_ *+ λ*_2_ + 1)*h* + *λ*_1_ + *λ*_2_ + 1} (*h* = 0,1,..,*m*_max_ − 1) for the antibody. One mutation point is randomly selected from these three regions, and the gene values of these genes are changed. A value is selected accordingly and randomly from the values of typical boarding stop numbers, typical alighting stop numbers and dense departure times in the vaccine, and these values must be replaced. The immunity process is shown in Fig 8.

The probability of immunity selection is set to a certain value *p*_*b*_; thus, antibodies that must implement the immunity selection operation based on *p*_*b*_ are randomly selected.

(11) Termination criterion of the IIGA

The termination criteria used in this paper is a certain number of iterations that determines a sufficiently large positive number T, and the total number of iterations for the algorithm does not exceed T. Due to the limited computation time and capacity, the number of iterations is not infinite. The advantages of this criterion are that the computation time can be easily controlled.

### 4.2 Flow of the IIGA

**Fig 9:**
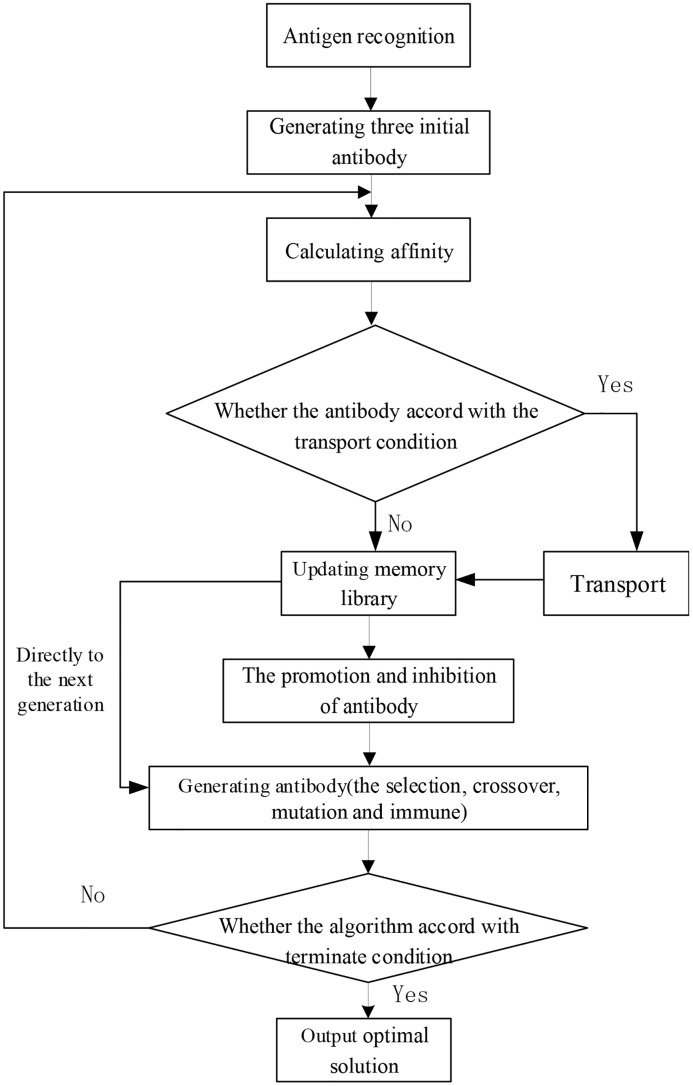
Flowchart of the IIGA.

The steps for designing the IIGA in this paper are as follows. The flowchart is shown in Fig 9.

Step 1. Antigen recognition. The problem of stop planning and timetable creation for CBs must be abstracted to the form of an antigen in accord with the behavior of the immune system, i.e., the problem is solved.

Step 2. Generate the initial antibody population. Three initial antibody populations are generated based on the principle of generating the initial antibody population and randomly generating the initial time in a reasonable range.

Step 3. Calculate the affinity. The affinity between the antibody and antigen in each subpopulation is calculated.

Step 4. Determine whether the antibody is consistent with the transport condition. If the antibody is consistent with the transport condition, the transport operator must implement the transport operation according to the algorithm. Otherwise, advance to Step 5.

Step 5. The antibody population with better affinity is stored in the memory library of the subpopulations, and the antibody genes in the memory library are directly inherited by the next generation population.

Step 6. Promote and inhibit the antibody. The antibody concentration is calculated by calculating the affinity between antibodies; the antibody viability is then calculated.

Step 7. According to the design of the algorithm, the antibody performs the selection, crossover, mutation and immune operations, and the antibody is updated.

Step 8. Determine whether the algorithm is consistent with the termination condition. If the algorithm is consistent with the termination condition, the optimal solution is the output. Otherwise, return to Step 2.

## 5 Case Study

Passenger travel demand is concentrated for Liyuan and Guomao. Liyuan covers an area of approximately 18 km^2^, and Guomao covers an area of approximately 3 km^2^. After demand acquisition, the demand is 568 in the morning peak period from Liyuan to Guomao; the travel densities in Liyuan and Guomao are 47 and 189 people/km^2^, respectively; and the travel demand is dense. After screening, these requirements are distributed to 57 residential districts in the Liyuan community and 57 office buildings in Guomao, which are scattered in the two traffic zones. The residents' expected workplace arrival time is 8:15, 8:30, 8:45, 9:00 or 9:15. According to the coding mode implemented in this paper, $t_{i}^{sd}\in \{135,150,165,180,195\}$.

Every passenger boarding time *t*_*s*_ is 5 s, the alighting time *t*_*x*_ is 5 s, and the necessary dwell time is 30 s. Passengers’ personal annual income *NC*(*i*) is 120,000 yuan, the annual number of working days is 250, the annual working time *NT*(*i*) is an average of 120,000 min according to 8 working hours per day, the loss factor of travel time *α*_*g*_(*i*) is 100%, the loss factor of arriving at the destination ahead of schedule *α*_*f*_(*i*) is 50%, and the proportion of passenger travel value and wages per unit time *α*_*x*_(*i*) is 80%.

According to Beijing CB fees, the travel fee consists of a seat fee and credit card fee for each take. The standard seat fee within 20 km (inclusive) is 8 yuan and increases by 3 yuan with each additional 5 km (inclusive). The average CB speed is 40 km, the driver fixed cost *C*_*pdg*_ is 50 yuan, the unit time cost of driving a vehicle is *ω*_*d*_ 1 yuan/min, the average passenger weight is 60 kg, the vehicle weight is 11,500 kg, the fuel cost of unit distance *ω*_*r*_ is 2.5×10^−4^ yuan/(km•kg), the vehicle purchase fee $C_{g}^{h}$ is 700,000 yuan, the vehicle service life *ε*^*h*^ is 8 years, the number of vehicle uses per day nch is 2, and the congestion cost of unit area *ω*_*yj*_ is 0.02.

Passenger boarding stop and alighting stop demand data are calculated by the K-means algorithm in MATLAB according to the distance between each boarding (alighting) stop. The clustering results meet the demand for a distance within 500 m because passengers can walk to alternate stops within this distance. After K-means clustering, 37 and 14 alternative stops are identified for Liyuan and Guomao, respectively. The location of the center of mass at which the clustering results are generated is an alternative stop. For the simple calculation, the alternative site number selects the original stop number that is closest to the center of mass, which is no longer renumbered.

### 5.1 Solution to the example using the IIGA

The parameters of the model and algorithm are set as shown in Table 1. The range of the crossover probability is [0.48, 0.9), and the range of the mutation probability is (0, 0.1].

**Table 1:** Parameters of the model and IIGA.

Parameter	Value	Parameter	Value	Parameter	Value	Parameter	Value
Population size M	100	*λ*_5_	20	*γ*_1_	5	*γ*_8_	10
Maximum genetic algebra T	500	*λ*_6_	80	*γ*_2_	5	*γ*_9_	3
Memory library size J	20	*λ*_7_	3	*γ*_3_	5	*α*_*rx*_	1.5
*λ*_1_	9	*λ*_8_	3	*γ*_4_	3	*k*_1_	1.8
*λ*_2_	9	*λ*_9_	15	*γ*_5_	1.1	*k*_2_	0.2
*λ*_3_	10	*λ*_10_	15	*γ*_6_	0.9	-	-
*λ*_4_	50	*pc*	1×10^6^	*γ*_7_	1	-	-

The optimal solution of the model is obtained by the IIGA as follows:

$$LX=\left[\begin{array}{cccccccccccccccccc} 54 & 50 & 50 & 47 & 36 & 37 & 37 & 38 & 38 & 111 & 106 & 106 & 106 & 85 & 85 & 68 & 68 & 68\\ 49 & 41 & 41 & 23 & 30 & 27 & 43 & 34 & 48 & 109 & 109 & 101 & 101 & 101 & 80 & 80 & 80 & 80\\ 22 & 25 & 19 & 30 & 11 & 10 & 6 & 5 & 2 & 112 & 112 & 107 & 80 & 80 & 58 & 58 & 58 & 58\\ 53 & 53 & 51 & 26 & 26 & 39 & 15 & 15 & 14 & 105 & 105 & 105 & 96 & 96 & 88 & 88 & 88 & 88 \end{array}\right]$$

**Fig 10:**
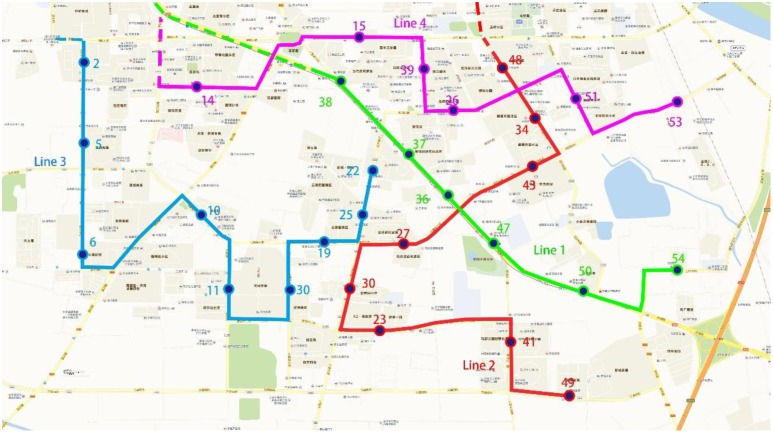
Stop distribution for boarding zones based on the IIGA.

**Fig 11:**
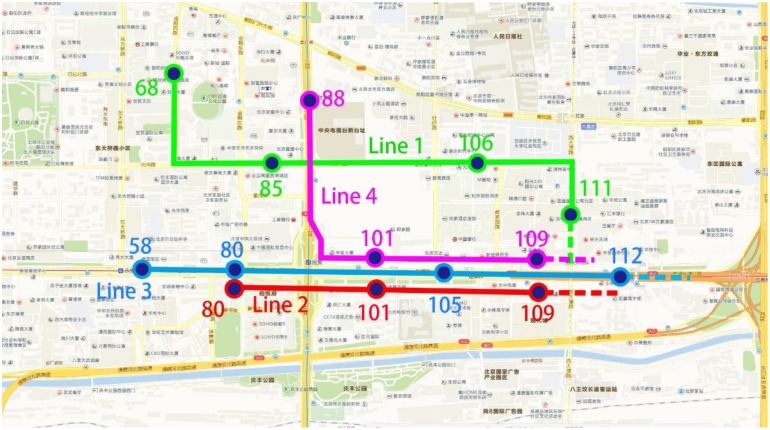
Stop distribution for alighting zones based on the IIGA.

$t_{1}^{s1}=114$, $t_{1}^{s2}=95$, $t_{1}^{s3}=90$, $t_{1}^{s4}=96$. The timetable in which the vehicle arrives at each stop can be calculated. The four CB lines are shown on the map, and the distributions of the boarding stops and alighting stops on each line are as shown in Figs 10 and 11, respectively.

The first CB boarding stops are stops 54, 50, 47, 36, 37 and 38, and the alighting stops are stops 111, 106, 85 and 68. For line 1, the stop ID, arrival time, number of boarding and alighting stops and other information are shown in Table 2.

**Table 2:** Vehicle driving information for Line 1.

Stop property	Stop number	The arrival time	Travel distance (meter)	Number of people boarding and alighting
Boarding stop 1	54	7:54	-	6
Boarding stop 2	50	7:57	1200	7
Boarding stop 3	47	8:01	1000	3
Boarding stop 4	36	8:03	654	5
Boarding stop 5	37	8:05	500	15
Boarding stop 6	38	8:09	887	5
Alighting stop 1	111	8:41	17000	10
Alighting stop 2	106	8:44	925	15
Alighting stop 3	85	8:48	948	11
Alighting stop 4	68	8:52	1100	5

Similarly, the boarding stops, alighting stops and relevant information can be drawn for the second, third and fourth CBs.

Passenger information and vehicle information are collected for four lines, as shown in Table 3.

**Table 3:** Vehicle running information based on the IIGA.

Project	Vehicle 1	Vehicle 2	Vehicle 3	Vehicle 4	Total
Number of passengers (persons)	41	45	39	38	163
Travel kilometers (km)	24.8	25	25.9	25	100.7
Travel time (min)	58	63	60	59	240
Passenger time cost (yuan)	1943	2338	1937.5	1955	8173.5
Passenger travel time (yuan)	1421.6	1714.4	1352	1380.8	5868.8
Fare income (yuan)	453	479	443.2	427.4	1802.6
Driver cost (yuan)	108	113	110	109	440
Fuel cost (yuan)	86.6	88.8	89.6	86.1	351.1
Depreciation cost (yuan)	119.9	119.9	119.9	119.9	479.6
Societal benefits (yuan)	617.7	686.1	612.2	575.1	2491.1

### 5.2 Analysis and comparison of results

The results of the IIGA and IGA are compared in terms of the number of stops, number of passengers, vehicle driving distance, travel time, average passenger travel time, average passenger arrival time ahead of schedule, and total line revenue in Table 4.

**Table 4:** Results of the IGA and IIGA.

Index	Results of the IGA	Results of the IIGA
Number of boarding stops/number of alighting stops	22/10	29/11
Number of passengers (persons)	132	163
Vehicle driving distance (km)	99.5	100.7
Travel time (min)	224	240
Average passenger travel time (min)	43.2	45
Average passenger arrival time ahead of schedule (min)	11.2	10.3
Total line revenue (yuan)	498	718.3

The results of the comparison in Table 4 illustrate that for the IIGA relative to the IGA, the numbers of boarding stops and alighting stops increased by 7 and 1, respectively; the number of passengers increased by 31; the vehicle driving distance increased by 1.2 km; the travel time increased by 16 min; the average passenger travel time increased by 1.8 min; the average passenger arrival time ahead of schedule decreased by 0.9 min; and the total line revenue increased by 220.3 yuan. The increase in the numbers of boarding stops and alighting stops indicates that a CB line covers a larger area; therefore, the distance, travel time and average travel time of the vehicle increase. The increase in the number of passengers causes the total line revenue to increase. Moreover, the average passenger arrival time ahead of schedule meets the passenger demand.

**Fig 12:**
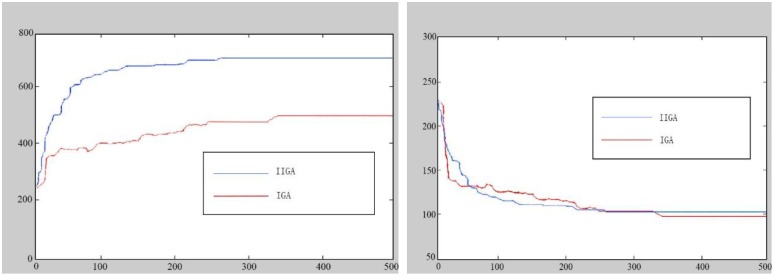
Graph of the algorithm convergence (a: Iteration diagram of total line revenue; b: Iteration diagram of vehicle travel distance).

In terms of the speed of the algorithm, the IGA required 532 s to complete, whereas the IIGA required 268 s. An iteration diagram of the total line revenue and vehicle travel distance is shown in Fig 12.

The IIGA also significantly outperforms the IGA in terms of the CB total line revenue. The IIGA shows advantages with respect to the number of stops, number of passengers, average passenger arrival time ahead of schedule and total line revenue, but the vehicle driving distance, travel time and average passenger travel time are inferior. In addition, the IIGA is significantly faster than the IGA, with a 49% faster running speed. Thus, the proposed model has strong potential for use in solving CB problems.

## 6 Conclusions

Based on an analysis of domestic and international research and the current development of CBs in China and considering passenger travel data, this paper studies the problems associated with the operation of CBs, such as stop selection, line planning and timetable setting, and establishes a model for the stop planning and timetable of CBs. The improved immune genetic algorithm (IIGA) is used to solve the model, which includes the following features: 1) multiple population design and transport operator design, 2) memory library design, 3) mutation probability design and crossover probability design, and 4) the fitness calculation of the gene segment. Finally, a real-world example set in Beijing is calculated, and the model and solution results are verified and analyzed. The results illustrate that the IIGA solves the model and is superior to the basic genetic algorithm in terms of the number of passengers, travel time, average passenger travel time, average passenger arrival time ahead of schedule and total line revenue. This study covers the key issues involving the operational system of CBs, combines theoretical research and empirical analysis, and provides a theoretical foundation for the planning and operation of CBs.

Future research will focus on the following aspects:

1. This paper does not consider the time delay caused by road congestion, and the arrival time of each stop is ideal. Future studies can combine road traffic flow data and arrival prediction theory to determine the exact arrival time of each stop, thus improving the punctuality rate and service level of CBs.
2. Because CBs are still in the initial stage of development for most cities and are simply attached to existing transit fleets, in this paper, the vehicle demands between two traffic zones optimize computation without considering fleet size restrictions. Therefore, in the future, the problem should be linked to fleet size, and relationships among travel demands, number of lines, boarding and alighting stops, departure times and fleet size should be considered.

## Supporting Information

S1 Dataset The passenger flow survey data. (XLSX)
